# Non-Invasive Longitudinal Bioluminescence Imaging of Human Mesoangioblasts in Bioengineered Esophagi

**DOI:** 10.1089/ten.tec.2018.0351

**Published:** 2019-02-14

**Authors:** Claire Crowley, Colin R. Butler, Carlotta Camilli, Robert E. Hynds, Krishna K. Kolluri, Sam M. Janes, Paolo De Coppi, Luca Urbani

**Affiliations:** ^1^ Stem Cells and Regenerative Medicine Section, UCL Institute of Child Health and Great Ormond Street Children's Hospital, University College London, London, United Kingdom.; ^2^ Lungs for Living Research Centre, UCL Respiratory, University College London, London, United Kingdom.; ^3^ Institute of Hepatology London, Foundation for Liver Research, London, United Kingdom.

**Keywords:** tissue engineering, transplantation, tissue scaffolds, bioluminescence

## Abstract

Esophageal engineering aims to create replacement solutions by generating hollow organs using a combination of cells, scaffolds, and regeneration-stimulating factors. Currently, the fate of cells on tissue-engineered grafts is generally determined retrospectively by histological analyses. Unfortunately, quality-controlled cell seeding protocols for application in human patients are not standard practice. As such, the field requires simple, fast, and reliable techniques for non-invasive, highly specific cell tracking. Here, we show that bioluminescence imaging (BLI) is a suitable method to track human mesoangioblast seeding of an esophageal tubular construct at every stage of the preclinical bioengineering pipeline. In particular, validation of BLI as longitudinal quantitative assessment of cell density, proliferation, seeding efficiency, bioreactor culture, and cell survival upon implantation *in vivo* was performed against standard methods in 2D cultures and in 3D decellularized esophageal scaffolds. The technique is simple, non-invasive, and provides information on mesoangioblast distribution over entire scaffolds. Bioluminescence is an invaluable tool in the development of complex bioartificial organs and can assist in the development of standardized cell seeding protocols, with the ability to track cells from bioreactor through to implantation.

**Impact Statement:**

Methodologies for incorporation of cells into tissue-engineered grafts, particularly at the later preclinical stages, are suboptimal and non-validated, and monitoring cell fate within scaffolds cultured in bioreactors and *in vivo* is challenging. In this study, we demonstrate how bioluminescence imaging (BLI) can overcome these difficulties and allow quantitative cell tracking at multiple stages of the bioengineering preclinical pipeline. Our robust bioluminescence-based approach allowed reproducible longitudinal monitoring of mesoangioblast localization and survival in 2D/3D tissue culture, in organ-scale bioreactors, and *in vivo*. Our findings will encourage the use of BLI in tissue engineering studies, improving the overall quality of cell–scaffold interaction research.

## Introduction

Esophageal tissue engineering aims to create a suitable replacement by following the principle of engrafting cells onto a tubular scaffold, creating a functional tissue or organ.^1^ A common aim in tissue engineering endeavors is the development of robust, controlled protocols for cell seeding and 3D culturing.^2,3^ While technology to seed cells onto scaffolds in controlled environments, or bioreactors, has developed rapidly, methods to monitor the fate of seeded cells have not kept pace.^4,5^ As such, tracking the engraftment, survival, and proliferation of cells in esophageal constructs from organ-scale bioreactor studies through to *in vivo* implantation represents a challenge for the field, where validation is necessary for clinical translation.^6,7^

The mainstay methods to image and/or quantify cells on tissue-engineered esophageal scaffolds include scanning electron microscopy, metabolic activity assays, DNA quantification assays, flow cytometry, confocal microscopy, and histochemistry. These techniques enable quantification and phenotypic analysis of seeded cells at a fixed time point but are limited by the requirement for termination of the experiment for analysis. Although technical replicates can be analyzed in parallel, longitudinal tracking of the same graft is not feasible. Moreover, these techniques limit analyses to small segments of grafts and cannot provide insight into the overall distribution of cells over the whole scaffold.

Bioluminescence imaging (BLI) has been used to perform real-time analysis of disease burden, track exogenous cells, and to determine the effectiveness of drugs, for example, in cancer studies.^8–10^ Cells are transfected with firefly luciferase, which catalyzes the oxidation of its substrate Luciferin—added to culture media at the time of imaging—to oxyluciferin, resulting in the release of energy in the form of light.^11^ A highly sensitive, cooled charged-coupled device camera allows non-invasive imaging of the luciferase signal. A number of characteristics of this system have enabled its utility in bioengineering studies.^12,13^ Firstly, only living, transduced cells can emit light because the luciferase reaction is ATP-dependent.^14^ Secondly, entire scaffolds can be analyzed simultaneously. Finally, the procedure is non-invasive, permitting real-time longitudinal monitoring of living cells in tissue culture, in *ex vivo* bioreactors, and *in vivo* at multiple time points.^15^

Mesoangioblasts are mesoderm-derived precursor cells, associated with small vessels and capillaries, and appear as a promising source of smooth muscle cells.^16^ In particular, we recently reported the use of human mesoangioblasts (hMABs) in the reconstruction of an esophageal *muscularis externa*, making these cells an attractive tool for the bioengineering of other visceral organs as well.^17^ One of the main challenges in the engineering of successful 3D organs is the optimization of robust methodologies. As part of our established culture system, here we validate tracking of primary hMABs in decellularized tubular esophageal scaffold at multiple levels of the preclinical tissue engineering workflow. We used transduced primary hMABs—derived from skeletal muscle—with a lentivirus carrying luciferase and the fluorescent protein ZsGreen, and compared BLI with established techniques for monitoring cells in 2D cultures such as MTT, which measures cellular metabolic activity, and CyQUANT, which measures the DNA content of cells. We further investigate cell seeding efficiency, proliferation, and migration in scaffolds in both static and dynamic 3D cell culturing conditions generated using a bioreactor described in our recent study in which we developed a multi-strata tubular esophageal substitute.^17^ Finally, we demonstrate that BLI allowed the tracking of cell engraftment, survival, and proliferation *in vivo* using a subcutaneous heterotopic xenograft model.

## Materials and Methods

### Stromal cell isolation and culturing

hMABs were isolated from skeletal muscle biopsies from pediatric patients, with informed consent, during operations at Great Ormond Street Hospital, London, in accordance with ethical approval by the NHS Research Ethics Committee (REC Ref: 11/LO/1522). The Committee was constituted in accordance with the Governance Arrangements for Research Ethics Committees and complied fully with the Standard Operating Procedures for Research Ethics Committees in the UK. Cells were isolated according to previously published protocol.^18^ Briefly, biopsies were dissected into small pieces (∼2 mm^3^), removing possible adipose tissue and seeded on petri dishes coated with Matrigel (growth factor reduced; BD Biosciences) diluted 1:100. Muscle fragments were covered with proliferation medium [Megacell medium (Sigma), 5% fetal bovine serum (FBS; Gibco), 1% non-essential amino acids (Gibco), 1% L-Glutamine (Gibco), 1% penicillin-streptomycin (Gibco), 0.1 mM beta-mercaptoethanol, and 5 ng/mL bFGF (Sigma Aldrich)] and incubated at 37°C, 5% O_2_, and 5% CO_2_. Cells were collected through trypsinization and passaged at 60–70% confluence for up to 10 passages.

### Lentivirus preparation

#### Lentivirus production

The lentiviral transfer vector pHIV-LUC-ZsGreen (Supplementary Fig. S1) was a gift from Dr. Bryan Welm (Department of Surgery, University of Utah, purchased through Addgene, Inc., MA, plasmid #39196) and was used to generate a lentivirus coding for ZsGreen florescent protein and firefly luciferase separated by an internal ribosome entry site, thereby enabling the two proteins to be translated from a single mRNA initiated by EF1-alpha promoter. Along with this third-generation lentivirus, we used the packaging plasmids pRSV-Rev (Addgene plasmid #12253) and pMDLg/pRRE (Addgene plasmid #12251) as well as the VSV-G envelope plasmid pMD2.G (Addgene plasmid #12259).

Briefly, lentiviral vectors were produced by co-transfecting 293T cells with the above plasmids.^19^ On day 1, 293T cells were plated in T175 flasks. On day 2, transfection was performed with cells at 70–80% confluence using a jetPEI (Polyplus Transfection) according to manufacturer's instructions. After 4 h of incubation at 37°C, the medium (complete DMEM, i.e., containing 10% FBS and penicillin/streptomycin; Gibco) was exchanged with fresh medium. On day 4 the medium was collected and replaced with fresh medium, which was also collected after a further 24 h. Virus-containing medium was purified by centrifugation at 2500 rpm (4°C) and filtered through a 0.45 μm membrane. Medium was ultracentrifuged at 18,000 rpm for 2 h at 4°C (SW28 rotor; Optima LE80K Ultracentrifuge, Beckham, High Wycombe). The viral pellet was resuspended in 100 μL precooled serum-free DMEM (Gibco), aliquoted, and the virus was stored at −80°C until use. Viral titers were calculated by transduction efficacy in HEK293T cells. Cells were expanded in complete DMEM and seeded at 5 × 10^4^ cells per well in a 24-well plate. A dilution series (1:5) from 20 μL/mL virus to 0.0032 μL/mL virus was created in a total volume of 500 μL per well. Cells were cultured overnight and changed for fresh medium the following day. Transduction efficacy was determined by flow cytometric analysis of the proportion of cells expressing the fluorescent protein ZsGreen 72 h after transfection. Viral titers were calculated from the volume of virus required to transduce cells between 10% and 20% efficacy according to the following formula^20–22^:

\begin{align*}
Viral { \rm { } } \ titer ( IU / mL ) { \rm { } } = { \frac { Number { \rm { } } \ of { \rm { } } \ cells { \rm { } } \ seeded \times percentage { \rm { } } \ of { \rm { } } \ florescent { \rm { } } \ positive { \rm { } } \ cells }  { Volume { \rm { } } \ of { \rm { } } \ virus { \rm { } } \ ( mL ) } } 
\end{align*}

#### Lentiviral transduction of stromal cells and fluorescence-activated cell sorting

hMABs were transduced with the lentivirus as described above but scaled to T25 flasks and tested at a variety of multiplicity of infections (MOI). Virus used at stock concentration was added in a dilutional series of 20, 10, 5, 1 μL per T25 flask. Transduction efficacy was determined by fluorescence-activated cell sorting (FACS) as a percentage of cells transduced (positive for ZsGreen). To obtain a pure population of transduced cells, cells were FACS-sorted following expansion of cells for one passage. Briefly, cells were trypsinized, centrifuged, and 1 × 10^6^ cells were resuspended in 500 μL of FACS buffer and sorted using FACSAria (BD Biosciences). Sorted cells were expanded for a further passage and checked by flow cytometry to ensure a pure population of transduced cells had been maintained and could be used for downstream experiments (Luc-ZsGreen^+^ hMABs).

### Decellularized scaffold preparation

Animal procedures were in accordance with ethical approval and UK Home Office Project License PPL 70/7622 and 70/7478. Sprague-Dawley male rats (200–300 g) were used for esophageal scaffolds. Donor tissue was harvested and trimmed and underwent wash steps in PBS. Esophagi were decellularized with two cycles of detergent-enzymatic treatment according to established protocols.^23,24^ Solutions were delivered dynamically through the esophageal lumen at 1 mL/min using a variable speed roller pump (*i*Pump). Decellularized esophagi were sterilized by gamma irradiation with 1.8 kGy.^17^

### Cell seeding in tubular decellularized scaffolds

Luc-ZsGreen^+^ hMABs were resuspended in a solution of sterile PBS, 0.1 ng/mL fibronectin (Sigma), and 0.5 ng/mL collagen I (Sigma), to obtain a final concentration of 1 × 10^6^ or 1.5 × 10^6^ cells/10 μL. Cells were kept on ice before seeding. The cell suspension was injected within the muscle wall of decellularized esophagi in multiple sites along three distinct longitudinal lines. One injection every 3–4 mm was performed using insulin syringes (MyJector) mounting 27G needles. The volume of cell suspensions was calculated to deliver 1 × 10^6^ or 1.5 × 10^6^ cells for every 5 mm scaffold length. Multiple microinjections were performed using a stereomicroscope located under a laminar flow cabinet. A nasogastric tube (6Fr; Enteral) was introduced within the lumen of tubular scaffolds to keep it under constant tension and to improve the ease of injections. The intrusion of the plastic tube caused removal of the esophageal mucosal layer. Finally, cell-seeded scaffolds were gently covered with proliferation medium and placed at 37°C. Samples for dynamic culturing were first incubated for 6 h in static culture to allow cell adherence to the esophageal matrix before being moved to the bioreactor.

### Cell tracking techniques

#### MTT colorimetric assay

The Vybrant MTT colorimetric assay (Life Technologies, Thermo Fisher) was used to assess cell viability and proliferation in 2D cultures, to determine cell engraftment and localization in 3D cultures and was performed according to manufacturer's instructions. Two-dimensional and 3D-cultured cells were incubated with MTT diluted 1:10 in proliferative medium for 4 h at 37°C. In 2D cultures, the reaction product, formazan, was solubilized in 100 μL of SDS and its concentration was determined by optical density at 550 nm. In 3D cultures, scaffolds were washed with PBS and imaged with a stereomicroscope.^17^ Formazan-positive hMABs were visible within the scaffolds.

#### CyQuant

CyQuant cell proliferation assay was performed to detect density of cells plated in 96-well plates, followed by storage at −80°C. Frozen plates were thawed to room temperature before adding 200 μL of CyQuant GR dye/lysis buffer, prepared following the manufacturer's instructions (Invitrogen). Plates were incubated in the dark for 5 min and fluorescence was measured at 485/535 nm using a Tecan plate reader.

#### Bioluminescent imaging

Bioluminescence was detected using an IVIS Lumina Series III Pre-Clinical In Vivo Imaging System (IVIS; Caliper Life Sciences) and Living Image 3.2 software (Caliper Life Sciences) as previously described.^10,17,20,25^ BLI was acquired as a measure of radiance. This is calculated from the number of photons emitted from the subject and recorded as the number of photons per second per cm^2^ per steradian (p/s/cm^2^/sr). All images were taken on either stage C or D, with the automated aperture setting, an automatic exposure time, and using a small binning (resolution).

##### Bioluminescent imaging of cell plates

Cells were imaged in 96-well plates to confirm luciferase expression and compare expression between populations of cells against standard pre-established protocols (MTT and CyQuant). Twenty minutes before imaging, culture medium was exchanged for medium containing 150 μg/mL D-Luciferin. An optimal time point, after which BLI had stabilized following the addition of D-Luciferin medium, was determined.

##### Bioluminescent imaging of 3D construct

Twenty minutes before imaging, fresh medium containing 150 μg/mL D-Luciferin was supplied to seeded scaffolds plated into multi-well dishes.

##### Bioluminescent imaging in a bioreactor

Culture medium containing 150 μg/mL D-Luciferin was injected into the internal chamber of the bioreactor via the three-way luer taps and imaged as described above. The bioreactor was placed on the stage and imaged using stage D for zoomed-out images of the entire reactor and stage C for all other images and analyses.

##### Bioluminescent image analysis

Images were analyzed using Living Image 3.2 software, generating pseudo-colored, scaled images overlaid on grey scale images, providing 2D localization of the source of light emission. Regions of interest (ROI) were selected using shape drawing tools, and light emission within the ROI was quantified in photons per second. ROI shapes were kept constant between subjects within each experiment.

### Subcutaneous implantation and bioluminescent imaging *in vivo*

Decellularized esophageal scaffolds with and without seeded Luc-ZsGreen^+^ hMABs were prepared as described above for *in vitro* experiments 24 h before implantation and submerged in medium at 37°C and 5% CO_2_. Adult NOD-SCID gamma (NSG) mice were used for subcutaneous implantation of scaffolds. Live animal work was ethically approved and carried out under Home Office Project License PPL 70/7504. Briefly, NSG mice were anesthetized with a 2–5% isoflorane:oxygen gas mix for induction and maintenance. Under aseptic conditions, the dorsum of the mouse was shaved and chlorhexidine was applied to the skin. A 3 mm transverse incision over each dorsal flank was made and a subcutaneous pocket created. One scaffold was inserted into each pocket such that there was an unseeded (control) scaffold on the left flank and a seeded scaffold on the right flank. Wounds were closed with 4/0 Vicryl interrupted, buried sutures.

Scaffolds were implanted for 7 days. Before imaging, 150 μL of 150 μg/mL D-Luciferin was injected into each scaffold directly. Images were acquired between 15 and 20 min after the addition of D-Luciferin as an initial time course experiment identified that this time was required for readings to stabilize. Mice were inducted and maintained with isoflurane:oxygen mix for imaging. Imaging was performed as for *in vitro* experiments but with the stage set to D and binning set to small. At the final time point, scaffolds were retrieved *en bloc*, washed in PBS, and fixed in 4% PFA at 4°C overnight for histopathological analysis.

### Immunohistochemistry

Fixed samples underwent standard processing for paraffin fixation. Serial paraffin sections were cut at 5 μm thickness. Tissue samples from *in vivo* implantation experiments were cut longitudinally along the whole length of the scaffold. Hematoxylin and eosin (H&E) staining was performed using an automated staining system (Tissue-Tek). hMABs were identified in murine xenograft experiments using an anti-human cell antibody, STEM121 (Clontech; Y40410; 1:1000), that identifies a cytoplasmic antigen present in human but not rodent cells, and an anti-luciferase antibody (Abcam; ab181640, 1:100). Detection was using a species-appropriate HRP-conjugated antibody, and the counterstain was hematoxylin.

### Epifluorescence

Samples were fixed in 4% PFA for 1 h at 4°C and then extensively washed in PBS. Dehydration was performed using solutions of progressively more concentrated sucrose. All samples were incubated with 10% and 15% sucrose for 30 min each and, finally, with 30% sucrose overnight at 4°C with slow agitation. Dehydrated tissues were embedded in O.C.T. (VWR) and immediately frozen by partial submersion in isopentane cooled in liquid N_2_. Finally, serial sections of 7 μm thickness were cut using a Leica cryostat and stored at −20°C. Frozen slides were thawed for 5 min at room temperature before being rehydrated in PBS. Tissue sections were permeabilized using 0.5% Triton X100 in PBS for 10 min at room temperature. Following washing with PBS, slides were mounted with DAPI (Abcam) and cover-slipped. A confocal microscope (Zeiss LSM710) was used to image the samples and identify nuclei (DAPI) and Luc-ZsGreen^+^ hMABs.

### Statistical analysis

Experiments were performed using mesangioblasts from one donor. Experiments were repeated on three separate occasions unless otherwise stated. Statistical analyses were performed using GraphPad Prism 7 software as described in figure legends. Statistical significance was assigned when *p* < 0.05.

## Results

### Cell transduction and monitoring in 2D cultures

**FIG. 1.**
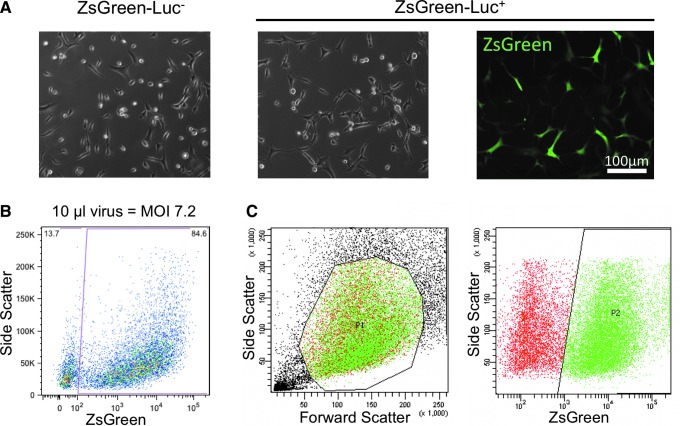
Purification of Luc-ZsGreen^+^ primary hMABs after effective transduction with ZsGreen-Luciferase lentiviral vectors. **(A)** Representative images of hMABs before (ZsGreen-Luc^−^) and after (ZsGreen-Luc^+^) transduction with lentivirus carrying the pHIV-Luc-ZsGreen vector (brightfield and ZsGreen fluorescence). **(B)** Transduction efficiency, determined by FACS analysis as a percentage of ZsGreen^+^ cells, using the MOI of 7.2, associated to the highest transduction efficiency, 84.6%. **(C)** ZsGreen-Luc^+^ cells were then sorted based on the expression of ZsGreen to obtain a purified population for seeding experiments. hMAB, human mesoangioblast; FACS, fluorescence-activated cell sorting; MOI, multiplicity of infections. Color images are available online.

Primary hMABs were transduced with a lentivirus carrying the pHIV-Luc-ZsGreen vector to express both ZsGreen fluorescent protein and luciferase. Following transduction, Luc-ZsGreen^+^ cells were morphologically indistinguishable from non-transduced cells (Fig. 1A). A MOI of 7.2 was selected as it was associated with the highest transduction efficiency (84.6%) as determined by FACS analysis (Fig. 1B). To avoid contamination with non-transduced cells, transduced hMABs were FACS-sorted based on the expression of ZsGreen (Fig. 1C) and further expanded in culture before seeding.

**FIG. 2.**
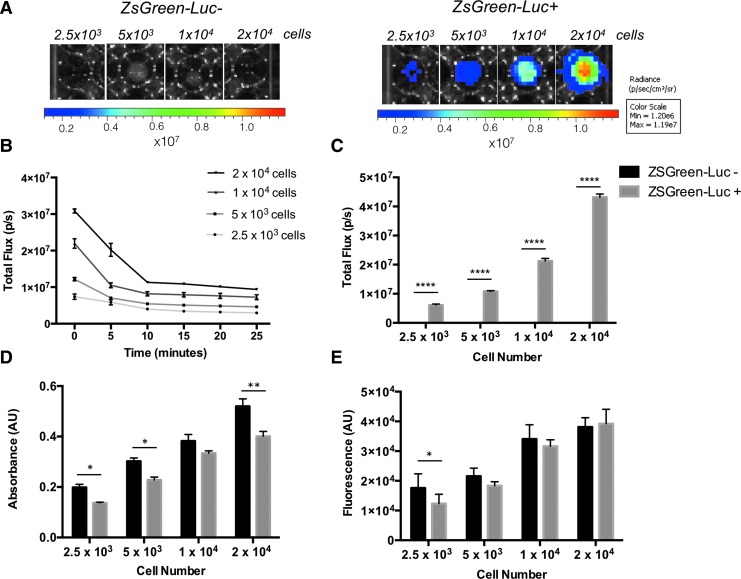
Bioluminescence effectively measures hMAB viability in 2D and discriminates between cell densities. **(A)** Bioluminescence images of Luc-ZsGreen^−^ and Luc-ZsGreen^+^ hMABs plated at different densities after 24 h of 2D culturing. **(B)** Graph of total flux measured every 5 min from different cell densities indicating the stabilization of bioluminescence over time. **(C)** Graph of bioluminescence total flux analyzed on different cell densities confirms bioluminescence signal is proportional to cell number and is negative for Luc-ZsGreen^−^ cells. Two-way ANOVA showed *p* < 0.001 for all possible comparisons of ZsGreen-Luc^+^ cell densities. No significant differences are detected for ZsGreen-Luc^−^ cells due to the absence of signal detected. **(D, E)** MTT and CyQuant assays also show that absorbance **(D)** and fluorescence **(E)** values correlate with cell number with a trend comparable to bioluminescence analysis. Data: mean ± SEM; *p < 0.05; **p < 0.01; ****p < 0.0001 (*n* = 3; two-way ANOVA). Color images are available online.

Purified Luc-ZsGreen^+^ hMABs and non-transduced Luc-ZsGreen^−^ control hMABs were seeded in multi-well plates at multiple densities (2500–20,000 cells per well of a 96-well plate) and proliferation was monitored using invasive (MTT; CyQuant) and non-invasive BLI techniques after 24 h of culturing. Transduced cells were detected using the IVIS upon exposure to D-Luciferin, while bioluminescence was not observed in non-transduced cells (Fig. 2A). To standardize bioluminescence detection, a time course analysis of total flux was performed at the same cell seeding densities. IVIS images were obtained at time 0 and every 5 min after the initial addition of 150 μg/mL D-Luciferin, and total flux was measured using the Living Image 3.2 software and the ROI tool. Bioluminescence showed an initial peak immediately after addition of D-Luciferin (time 0; Fig. 2B), and this was proportional to the number of cells seeded. Bioluminescence decreased rapidly to reach a plateau after 10–15 min post-D-Luciferin (Fig. 2B). This analysis provided a safe window for bioluminescence detection for subsequent experiments, in which bioluminescence readings were obtained 15–20 min after the addition of D-Luciferin to avoid errors due to the variability of readings between 0 and 10 min.

Quantification of the total flux emitted by Luc-ZsGreen^+^ hMABs cultured for 24 h showed a significant difference in bioluminescence between cell densities (two-way ANOVA; all comparisons *p* < 0.001; Fig. 2C), and as expected, non-transduced cells were negative for bioluminescence (Fig. 2C). We compared BLI with both MTT and CyQUANT, which are established invasive techniques traditionally used to determine cell number and viability in tissue culture applications. Both invasive assays showed comparable trends in cell viability at different cell densities with bioluminescence measures: absorbance (MTT; Fig. 2D) and DNA quantity (CyQUANT; Fig. 2E) increased in proportion to cell density. A small but significant difference in cell viability was detected between transduced and non-transduced hMABs using both MTT and CyQuant assays.

### BLI to monitor cells in 3D cultures

**FIG. 3.**
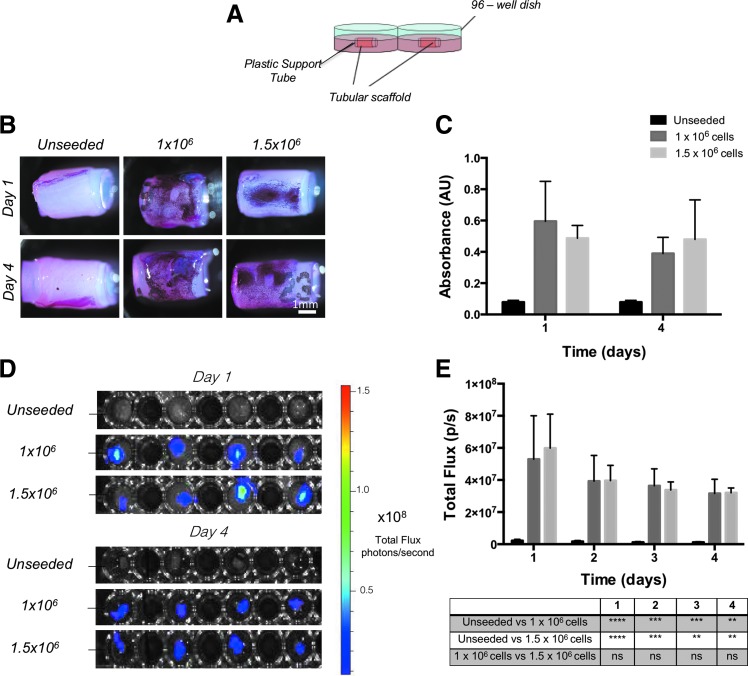
Bioluminescence imaging of 3D hMAB-seeded scaffolds for live cell viability detection in static cultures. **(A)** Five millimeter segments of unseeded and Luc-ZsGreen^+^ cell-seeded tubular scaffolds were cultured in static conditions in multi-well plates, with a plastic tube placed in the lumen to maintain the tubular structure throughout culturing. **(B)** MTT assays performed on unseeded or cell-seeded scaffolds cultured in static conditions for 1 or 4 days to visualize the cells and to determine cell viability. Two seeding densities (1 × 10^6^ or 1.5 × 10^6^) were tested. **(C)** Graph of absorbance from the extraction of formazan in the MTT assay showed no difference between the two seeding conditions (*n* = 3; *t*-test). **(D)** Bioluminescence imaging and **(E)** graphs of bioluminescence total flux performed daily for 4 days, showing comparable results to the MTT assay. Data: mean ± SEM (*n* = 3–4; two-way ANOVA). Color images are available online.

To monitor Luc-ZsGreen^+^ hMABs in a 3D context, they were injected in the wall of decellularized rat esophageal scaffolds obtained after two cycles of decellularization as previously described.^17^ Scaffold tubular structures were maintained throughout culturing by introducing a plastic tube into the lumen. Unseeded and scaffolds seeded with 1 × 10^6^ and 1.5 × 10^6^ cells per scaffold were cultured in static conditions in multi-well plates (Fig. 3A). Constructs were cultured for up to 4 days in static conditions. MTT assays performed on these scaffolds after 1 or 4 days of culturing were used to identify the location of cells within the scaffolds (Fig. 3B). From the quantification of formazan metabolized by the cells within the scaffold, no statistical difference was detected between the two cell densities nor the two time points (Fig. 3C). For this analysis, two separate sets of seeded/unseeded scaffolds were prepared since the MTT assay does not allow continuation of culturing. Unseeded scaffolds or those seeded with Luc-ZsGreen^+^ hMABs at cell densities of 1 × 10^6^ or 1.5 × 10^6^ cells per scaffold were also imaged daily with IVIS after the addition of D-Luciferin (Fig. 3D). BLI confirmed the absence of a significant difference between the two cell densities or in cell viability during culturing (Fig. 3E). The daily detection of bioluminescence allowed constant monitoring of the same constructs without stopping individual cultures.

### BLI of seeded tubular scaffolds cultured in a bioreactor

**FIG. 4.**
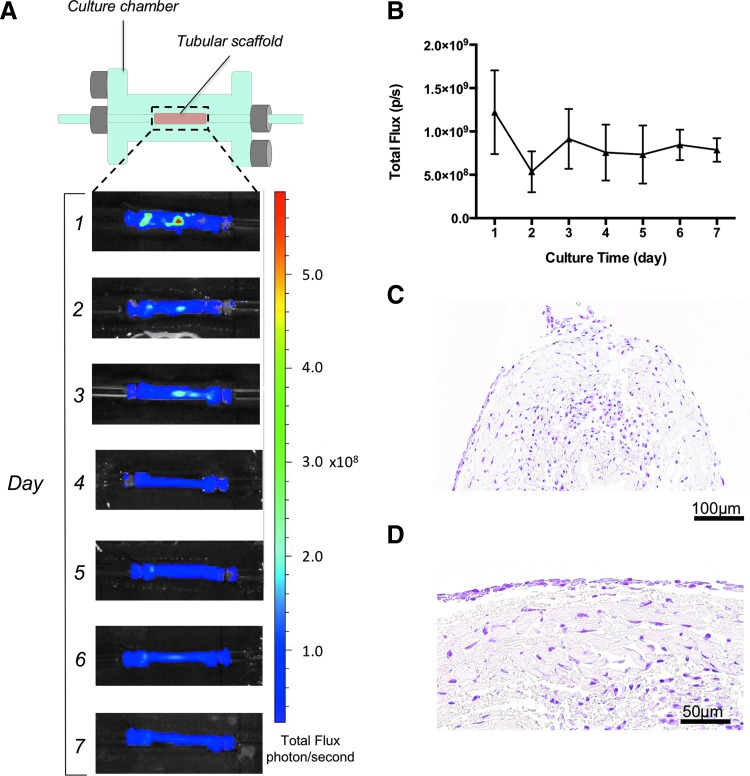
Bioluminescence imaging of 3D hMAB-seeded scaffolds in a dynamic cell culturing bioreactor. **(A)** Schematic of the dual glass culturing chamber for tubular scaffolds (*top*) and bioluminescence imaging of a representative cell-seeded scaffold placed in the glass chamber and cultured for 7 days (*bottom*). **(B)** Graph of bioluminescence total flux shows stabilization of cell viability during the culturing period. Data: mean ± SEM (*n* = 3). **(C, D)** Representative images of H&E staining to confirm the presence of cells at the end of culturing. The same area is shown at low **(C)** and high **(D)** magnification. H&E, hematoxylin and eosin. Color images are available online.

Static 3D tissue-engineered organ cultures do not incorporate compartmentalization, rotation, or flow^26^ and as such are suboptimal to achieve even cell distribution across scaffolds. Therefore, we tracked cells seeded onto scaffolds and cultured in a bioreactor that incorporated flow. Tubular scaffolds seeded with 1 × 10^6^ cells per 5 mm scaffold were cultured in a custom-made glass dual chamber to allow medium perfusion through the lumen. The external surface of the scaffold was immersed in medium contained in the external chamber (Fig. 4A). Medium flow through the lumen was controlled by a peristaltic pump. Bioluminescence of Luc-ZsGreen^+^ hMABs was measured every 24 h after the addition of D-Luciferin. The use of a glass chamber with no opaque components prevented interference problems, and cell distribution and viability was determined from IVIS images (Fig. 4A, B). During the experiment, cells progressively moved along the scaffold length from a clustered distribution, corresponding to the injection sites, to reach an overall homogeneous distribution. An initial decrease in cell growth 2 days post-seeding was followed by a subsequent stabilization of the cultures (Fig. 4B). At the end of culturing, cell-seeded scaffolds were processed for histology. Serial sections of paraffin-embedded samples stained with H&E confirmed the presence of hMABs throughout the length of the scaffolds and showed that these were distributed among the several layers of matrix (Fig. 4C, D).

### BLI of the engineered construct *in vivo*

**FIG. 5.**
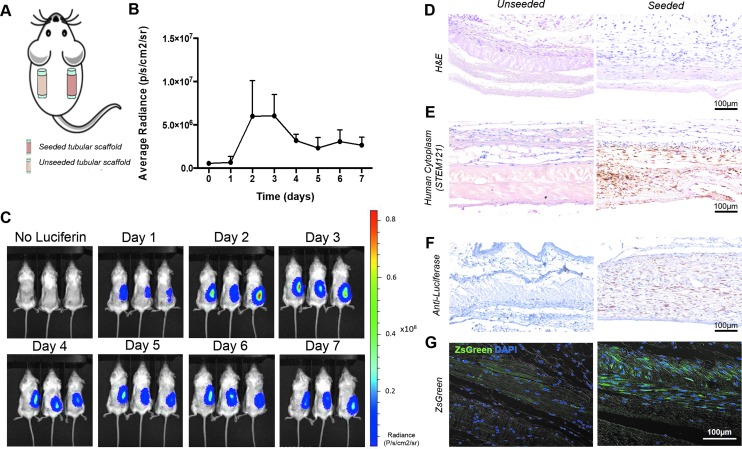
Bioluminescence imaging of 3D hMAB-seeded scaffolds *in vivo*. **(A)** Unseeded and Luc-ZsGreen^+^ hMAB-seeded scaffolds were implanted subcutaneously in the flank of NSG mice. **(B)** Graph of bioluminescence average radiance shows quantification of transplanted cells during the 7-day *in vivo* analysis. Data: mean ± SEM (*n* = 3). **(C)** Daily bioluminescence imaging of NSG mice with implanted unseeded and Luc-ZsGreen^+^ cell-seeded scaffolds. **(D–G)**. **(D)** H&E, **(E)** representative images of immunohistochemistry using STEM121, **(F)** anti-luciferase, and **(G)** ZsGreen epifluorescence to demonstrate the presence of Luc-ZsGreen^+^ hMABs in cell-seeded scaffolds after 7 days *in vivo*, while no signal was detected in unseeded control scaffolds. NSG, NOD-SCID gamma. Color images are available online.

Having demonstrated the compatibility of BLI with cell culture in a clinically relevant bioreactor setup, we sought to track Luc-ZsGreen^+^ hMABs on a scaffold implanted *in vivo*. Decellularized esophageal scaffolds were cell-seeded and implanted into the subcutaneous space on the back of immunocompromised NSG mice. A matched unseeded scaffold was implanted on the contralateral flank of each mouse as a control (Fig. 5A). Animals were imaged with IVIS every 24 h. Bioluminescence from Luc-ZsGreen^+^ hMAB-seeded scaffolds peaked 2–3 days post-implantation, then decreased to a stable plateau for 7 days (Fig. 5B, C). Unseeded scaffolds never showed any signal after injection of D-Luciferin, demonstrating that no cell migration from one scaffold to the other occurs in our model (Fig. 5C). H&E staining of paraffin-embedded scaffolds harvested after 7 days showed mild tissue remodeling and little inflammation (Fig. 5D). Cells were present around and inside the scaffold following the architecture and orientation of its native extracellular matrix (Fig. 5D). Immunohistochemistry using STEM121, an antibody that targets a cytoplasmic protein expressed specifically in human cells, and an anti-luciferase antibody confirmed the specificity of BLI cell tracking for transplanted cells as these were only present within seeded scaffolds (Fig. 5E, F). ZsGreen is brighter and more resistant to fixation than EGFP,^27^ so the presence of Luc-ZsGreen^+^ hMABs was also confirmed by fluorescence imaging of dewaxed, DAPI counter-stained sections (Fig. 5G).

## Discussion

Adequate tracking of cells in tissue engineering is essential in determining optimal scaffold conditions and cell engraftment strategies. Typically, methods require the termination of the experiment, either through histology or through destructive assays to quantitate cell engraftment. In other techniques, there is a reliance on the use of tracer agents, which are subject to interference from the scaffold itself or will decay over time, preventing reliable long-term tracking of cells. As such, numerous different experimental endpoints have to be considered to determine optimal regenerative conditions. Here, we used BLI to track primary hMABs spatially and longitudinally in a decellularized esophageal construct at each stage of the preclinical tissue engineering process. Importantly, this has been rigorously validated with methods considered standard for evaluating cell engraftment in tissue-engineered constructs.

While BLI has been used extensively to monitor developmental processes,^28^ infection,^29–31^ cancer,^10,32^ exogenous cell-mediated^8,33–36^ or extracellular vesicle-mediated^37^ therapies, its uptake in bioengineering has been quite limited.^17,38–45^ *In vitro* studies showed that BLI can be used to track the expansion of luciferase-transduced human mesenchymal stem cells on biomaterials^39^ and to assess the sheer force effects of perfusion bioreactors on cell distribution.^43^ Moreover, prior *in vivo* work has used BLI to monitor the cellular response to wounding and to foreign materials. Primary murine bone marrow-derived mononuclear cells co-expressing luciferase and GFP were tracked following tail vein injection into mice that had subcutaneous wounds or transplanted biomaterials.^42^ Additionally, BLI allowed monitoring of: mouse embryonic fibroblasts implanted subcutaneously within synthetic hydrogel or coral scaffolds,^39^ of mesenchymal stem cells on scaffolds implanted subcutaneously^41^ or in bone/spinal injury models,^38,44^ and of cardiomyocytes transplanted within a prevascularized gelatin scaffold following myocardial infarction.^40^ These studies showed that BLI can be effective in monitoring the regenerative response. We expanded these findings to hMABs—which have been proved to be an effective source of smooth muscle cells—seeded into decellularized tubular scaffolds. Notably, we validated BLI against established but invasive techniques, proving that BLI is a valuable tool for monitoring hMAB behavior during the culturing period and *in vivo*.

Since the bioengineering of complex tissues and organs is likely to involve multiple primary human cell types, the BLI method has a further advantage in that multiple luciferases can be used to track multiple cell types simultaneously. Reporters can be placed under the control of inducible tissue-specific promoters to monitor cell differentiation,^43,45^ and luciferases that luminesce at different wavelengths can be used in the same cell to track cell differentiation in scaffolds as well as survival and proliferation.^46^ Such techniques allow the study of multiple cell behavior in 3D constructs, a degree of complexity beyond the reach of other preclinical tissue engineering approaches.

For bioreactor research, moving beyond very low throughput is a challenge in a research laboratory: the chambers are costly, high cell numbers are required, and incubator space for bioreactors is limited. BLI, however, is relatively inexpensive and provides a convenient way to maximize the use of bioreactors by reducing the number of reactors that are necessary to run in parallel. Indeed, a recent report showed that it is possible for BLI to be inbuilt into the bioreactor setup to allow continuous monitoring of cells.^43^ Additionally, homogenous cell distribution after seeding in bioengineered scaffolds is key for graft functionality, as empty pockets would likely result in necrotic areas. BLI allows the localization of cells within whole scaffolds, so appropriate distribution, as well as number, can be verified. Importantly, while we used rodent scaffolds in the present study, the IVIS setup is compatible with bioreactors for human-sized organs. We foresee that BLI will be a useful tool for determining optimal human scaffold seeding protocols and monitoring cellular dynamics in bioreactor conditions.

There remain a number of questions around the fate of transplanted cells in tissue-engineered organ replacements that might be addressed by the compatibility of BLI with *in vivo* investigations,^47,48^ and here we showed the application of the technology to track primary human cells on biological, tubular tissue-engineered scaffolds. Since BLI can image cells at depths greater than those demanded by subcutaneous implantation, we envisage its application in experiments involving orthotopic tubular organ transplantation. A challenge remains, however, to develop imaging systems capable of monitoring cells in larger animal models, which are needed as orthotopic organ replacement is very challenging for some rodent organs. The ability to monitor cells in living animals also offers an opportunity to reduce the number of animals used in tissue engineering research.^49^

## Conclusion

Our study suggests that stable transduction of hMABs with a luciferase-containing multimodal reporter virus will be a valuable tool during the translation of esophageal bioengineering approaches. Transduced hMABs behaved similarly to non-transduced controls, but their location, engraftment, proliferation, and migration could be longitudinally monitored during experiments that span much of the preclinical bioengineering pipeline, from simple *in vitro* studies, through 3D organ-scale cultures to small animal models.

## Supplementary Material

Supplemental data
