# Supplementary material for: Non-Invasive Longitudinal Bioluminescence Imaging of Human Mesoangioblasts in Bioengineered Esophagi
Source: Tissue Eng Part C Methods. 2019 Feb 14;25(2):103–13. doi: 10.1089/ten.tec.2018.0351 (PMC6389770; doi:10.1089/ten.tec.2018.0351)
Supplement: Supplemental data [file Supp_Fig1.pdf]

Supplementary Data

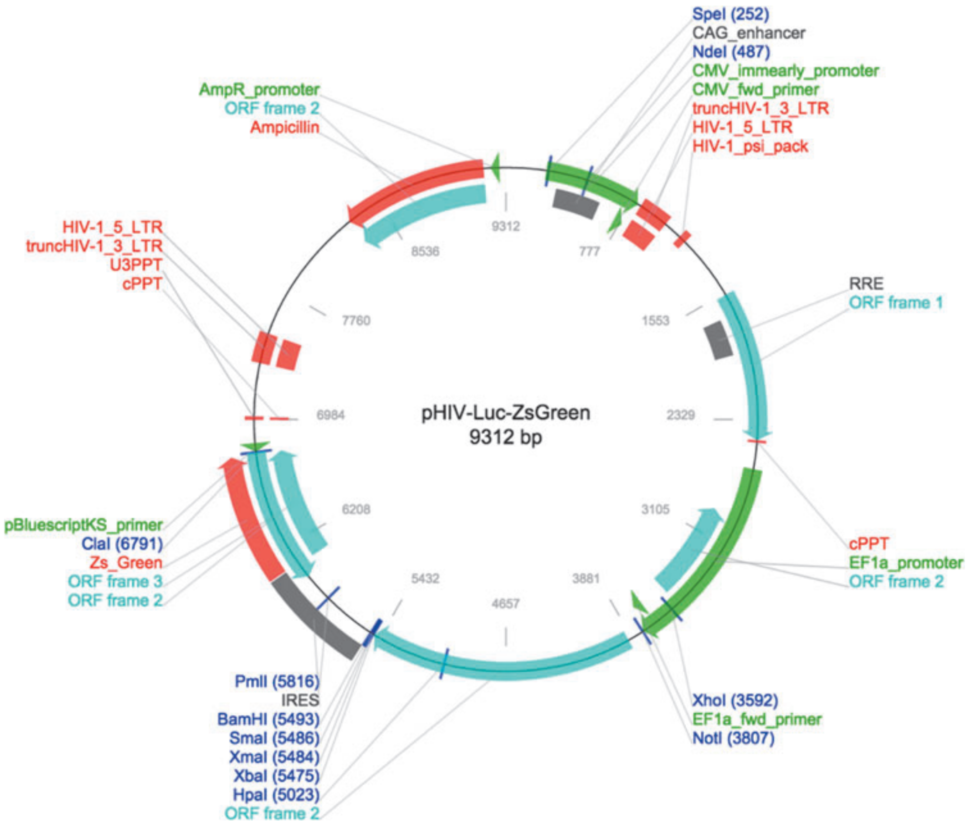

SUPPLEMENTARY FIG. S1. The lentiviral transfer vector pHIV-LUC-ZsGreen. The lentivirus coding for ZsGreen florescent protein and firefly luciferase separated by an IRES enables the two proteins to be translated from a single mRNA initiated by EF1-alpha promoter. IRES, internal ribosome entry site.
